# Supplementary material for: Analysis of Non-Polar Low-Molecular Metabolites in Citron (Citrus medica L.) Peel Essential Oil at Different Developmental Stages and a Combined Study of Transcriptomics Revealed Genes Related to the Synthesis Regulation of the Monoterpenoid Compound Nerol
Source: Int J Mol Sci. 2025 Sep 17;26(18):9034. doi: 10.3390/ijms26189034 (PMC12470865; doi:10.3390/ijms26189034)
Supplement: Supplementary file 1 [file ijms-26-09034-s001.zip › Figure S3.pdf]

Note: All special symbols appearing in this document have no additional meanings. For example, the special symbols such as * in "***** Results: *****" carry no special significance and are only used as eye-catching markers to remind readers to check the content.

BUSCO version is: 5.5.0
The lineage dataset is: embryophyta_odb10 (Creation date: 2024-01-08, number of genomes: 50, number of BUSCOs: 1614)
Summarized benchmarking in BUSCO notation for file /mnt/hgfs/Unigene.fasta
BUSCO was run in mode: euk_tran

***** Results: *****

C:79.8%[S:78.7%,D:1.1%],F:11.2%,M:9.0%,n:1614

1288 Complete BUSCOs (C)

1270 Complete and single-copy BUSCOs (S)

18 Complete and duplicated BUSCOs (D)

181 Fragmented BUSCOs (F)

145 Missing BUSCOs (M)

1614 Total BUSCO groups searched

Dependencies and versions:

hmmsearch: 3.1

metaeuk: 7.bba0d80

busco: 5.5.0

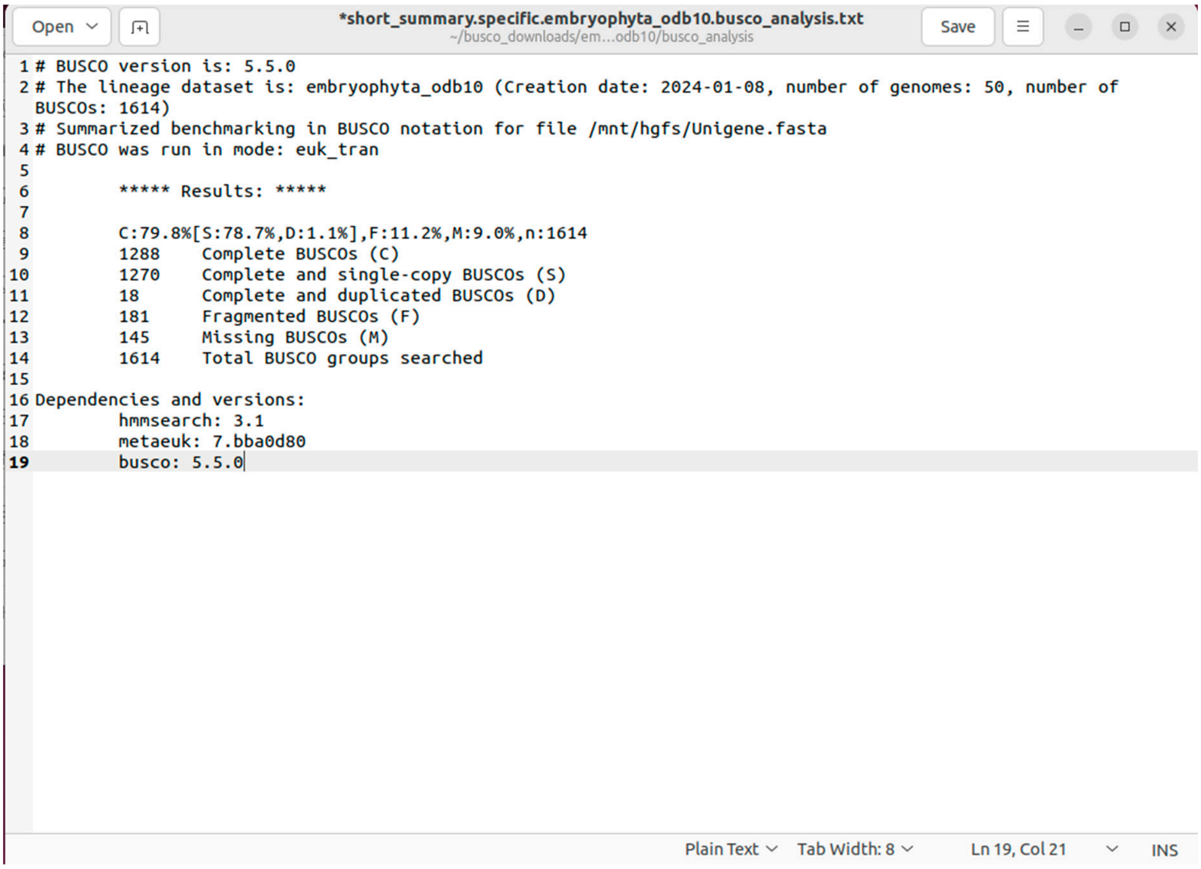


```
# BUSCO version is: 5.5.0
# The lineage dataset is: embryophyta_odb10 (Creation date: 2024-01-08, number of genomes: 50, number of BUSCOs: 1614)
# Summarized benchmarking in BUSCO notation for file /mnt/hgfs/Unigene.fasta
# BUSCO was run in mode: euk_tran

        ***** Results: *****

        C:79.8%[S:78.7%,D:1.1%],F:11.2%,M:9.0%,n:1614
        1288    Complete BUSCOs (C)
        1270    Complete and single-copy BUSCOs (S)
        18      Complete and duplicated BUSCOs (D)
        181     Fragmented BUSCOs (F)
        145     Missing BUSCOs (M)
        1614    Total BUSCO groups searched

Dependencies and versions:
        hmmsearch: 3.1
        metaeuk: 7.bba0d80
        busco: 5.5.0
```

BUSCO plot

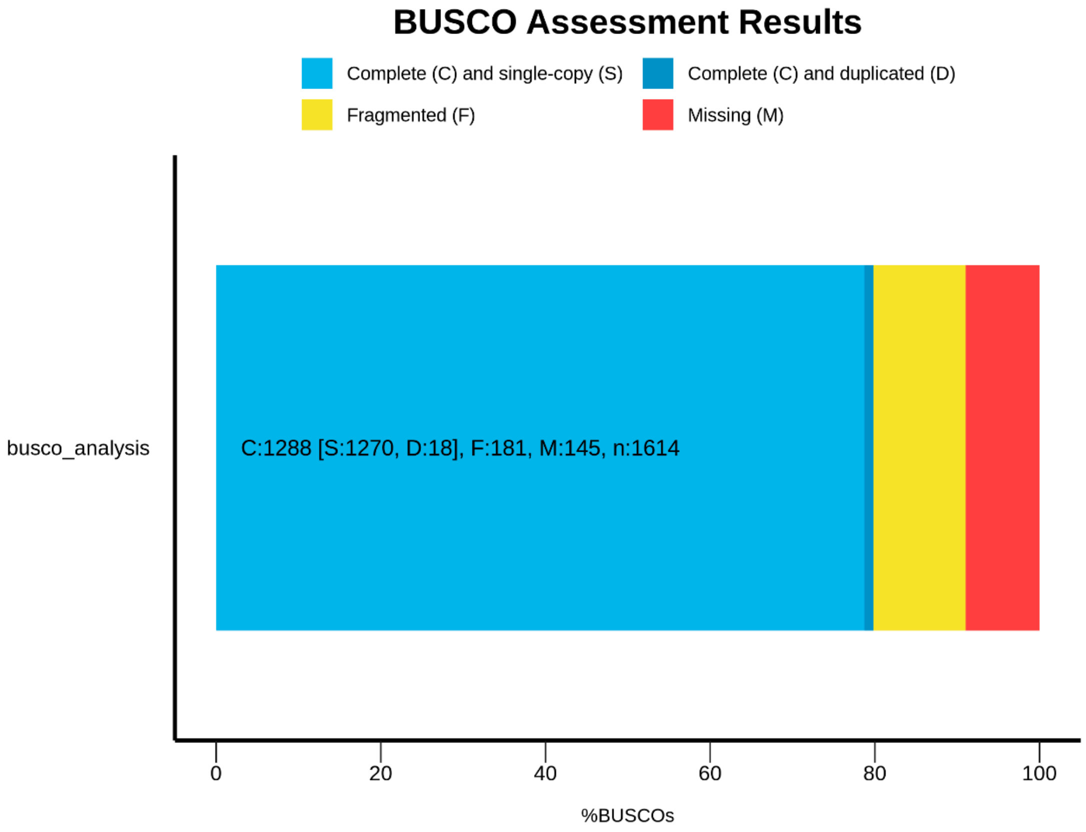

BUSCO Assessment Results
Complete (C) and single-copy (S)
Complete (C) and duplicated (D)
Fragmented (F)
Missing (M)
busco_analysis
C:1288 [S:1270, D:18], F:181, M:145, n:1614
0
20
40
60
80
100
%BUSCOs
