# Supplementary material for: Analysis of Non-Polar Low-Molecular Metabolites in Citron (Citrus medica L.) Peel Essential Oil at Different Developmental Stages and a Combined Study of Transcriptomics Revealed Genes Related to the Synthesis Regulation of the Monoterpenoid Compound Nerol
Source: Int J Mol Sci. 2025 Sep 17;26(18):9034. doi: 10.3390/ijms26189034 (PMC12470865; doi:10.3390/ijms26189034)

PCA Plot of Unigenes

seqQF_*: transcriptome samples at the green fruit stage

seqQH_*: transcriptome samples at the green-yellow fruit stage

seqYF_*:transcriptome samples at the yellow fruit stage

*: the asterisk (*) represents any digit.

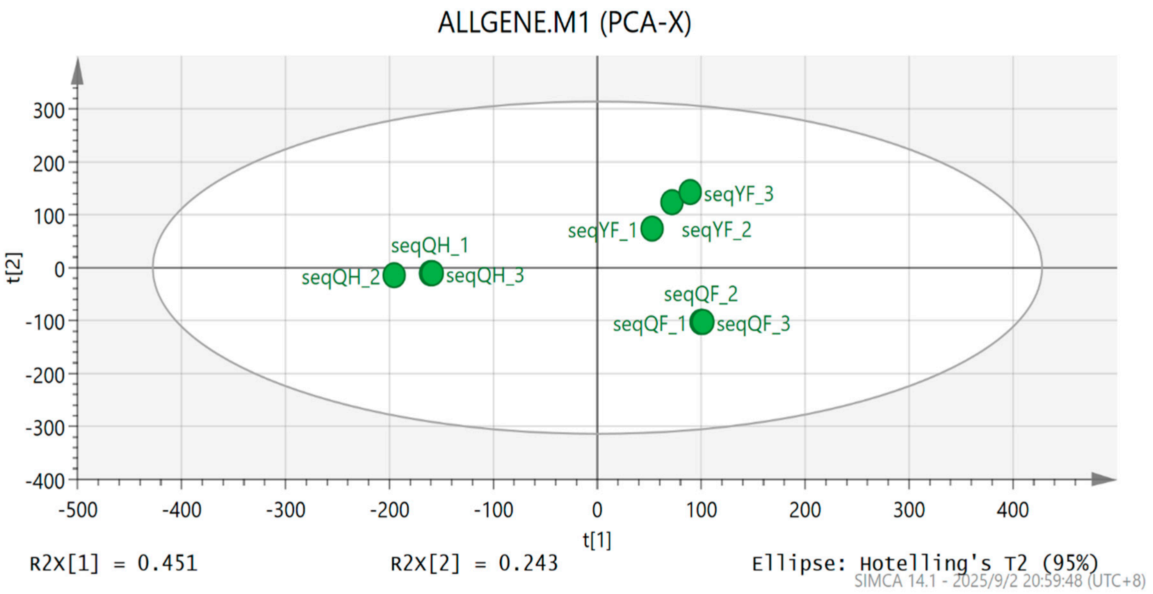

Supplement: Supplementary file 1 [file ijms-26-09034-s001.zip › Figure S4.pdf]
