# Supplementary figures and images for: Analysis of Non-Polar Low-Molecular Metabolites in Citron (Citrus medica L.) Peel Essential Oil at Different Developmental Stages and a Combined Study of Transcriptomics Revealed Genes Related to the Synthesis Regulation of the Monoterpenoid Compound Nerol
Source: Int J Mol Sci. 2025 Sep 17;26(18):9034. doi: 10.3390/ijms26189034 (PMC12470865; doi:10.3390/ijms26189034)

Total ion current chromatograms detected in each period:

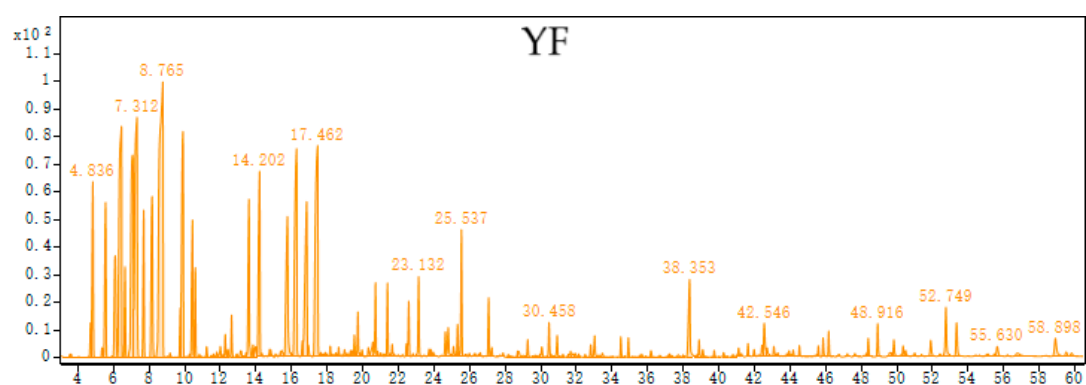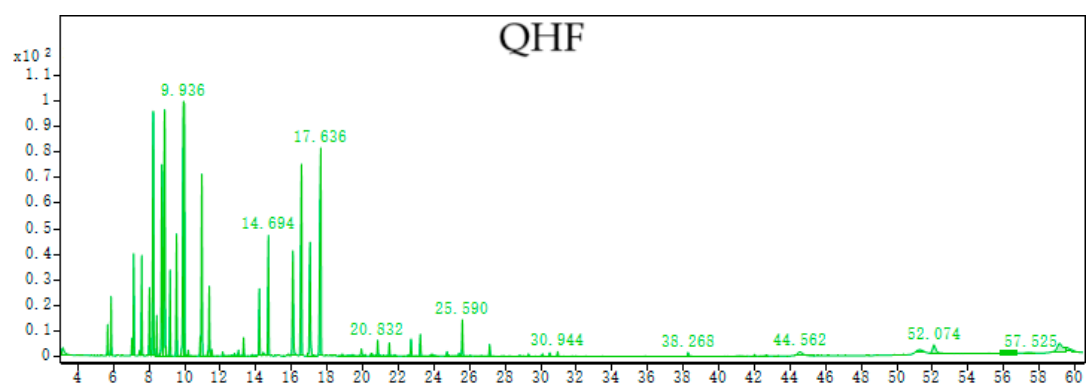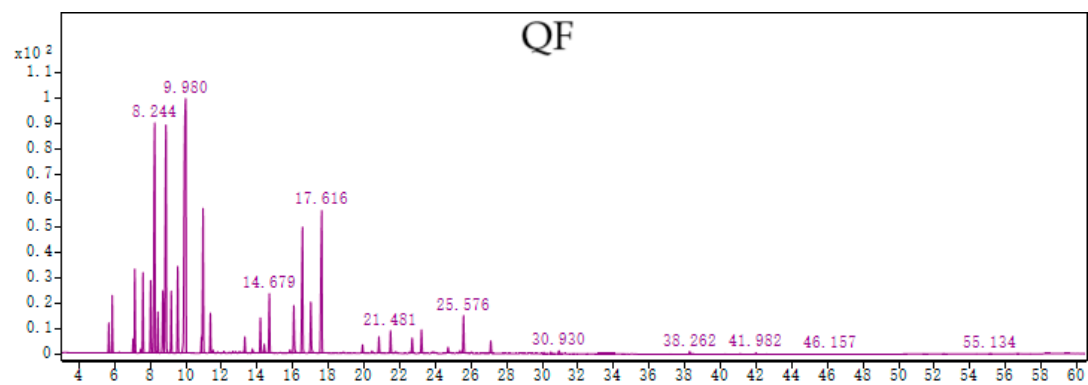

Supplement: Supplementary file 1 [file ijms-26-09034-s001.zip › Figure S1.pdf]

PCA sample scores plot:

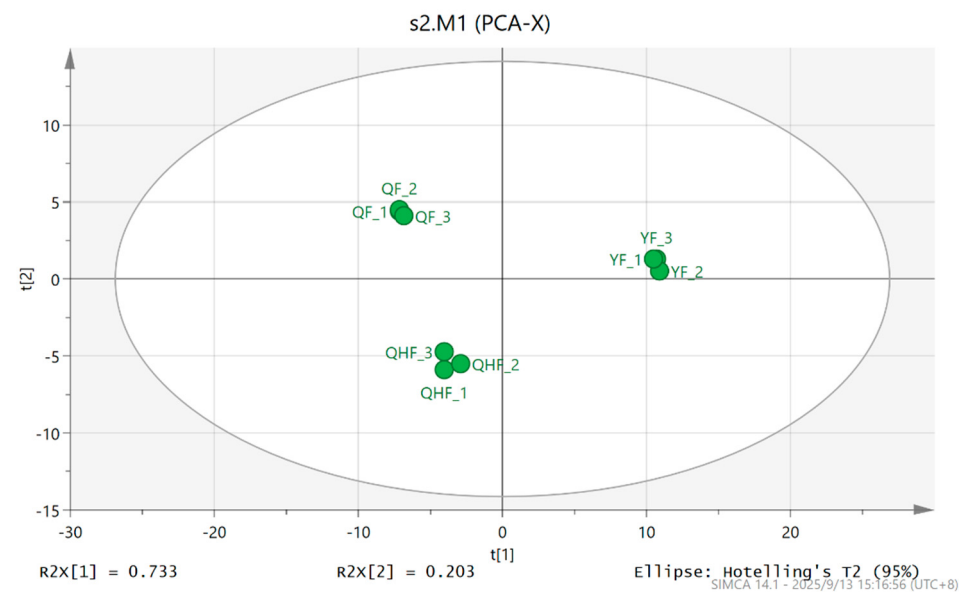

PCA x-loadings plot:

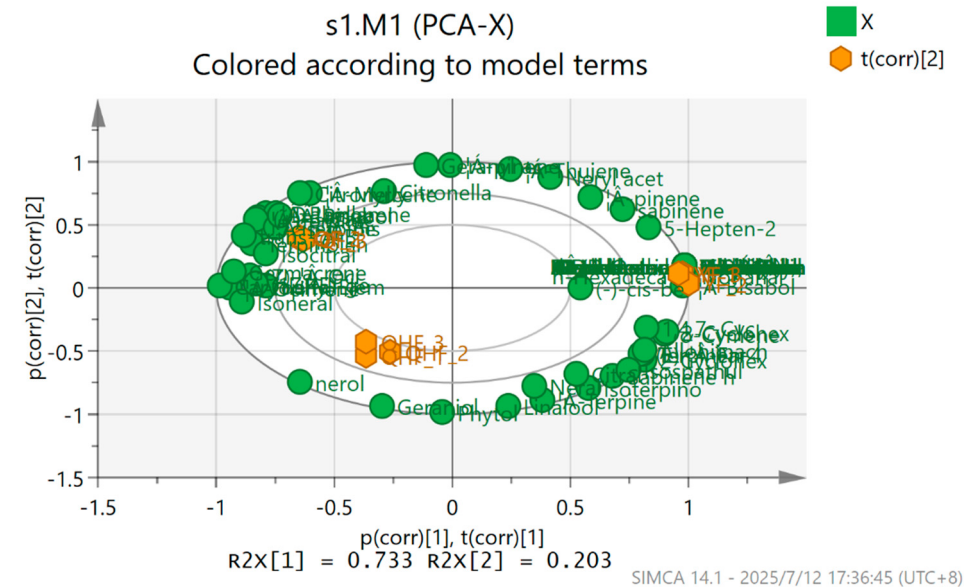

Supplement: Supplementary file 1 [file ijms-26-09034-s001.zip › Figure S2.pdf]
